# Acetate of Lead in Diseases of the Mucous Membranes

**Published:** 1834

**Authors:** 


7. *Acetate of Lead in diseases of the Mucous Membranes.*—An anonymous writer in the *Boston Journal*, has the following observations on the powers of this preparation of lead, in certain morbid states of the mucous membranes. We are the more disposed to present them to our readers, from having, at this moment under our care, a dysenteric patient, who is deriving great advantage from the acetate of lead and opium, after all the ordinary remedies had entirely failed.

"The most important known property of lead is its power to relieve certain kinds of inflammation and irritation of the mucous membranes, accompanied by profuse or otherwise morbid secretions. It seems to have a specific operation upon the capillary vessels, removing congestion, allaying excitement and disordered action, and restoring the balance between the secernent and absorbent vessels. It reduces frequency and hardness of pulse when dependent upon this state of the tissues, particularly the mucous tissues, calms the system and quiets febrile irritation. It does not seem to have any very positive direct action upon the sanguineous system; hence it may be used in quite different states of this system. It may be employed in chronic, acute, and subacute diseases, when the case is appropriate. I have employed it in several cases of acute typhoid pneumonia, particularly in its second stage, accompanied by profuse muco-sanguineous expectoration, distressed respiration, frequent, irritated pulse, hot or sweaty skin, &c., somctimes with very remarkable success. In such cases it should be employed very freely, say from two to five grains every two or four hours, in combination with opium. Generally the teeth and gums become of a leaden color after using it thus freely twenty-four or forty-eight hours, which is as long a time ordinarily as is necessary. In chronic, irritative coughs, without secretion from the membrane of the lungs, and in coughs attended with copious secretion, I have found it an invaluable remedy, combined with opium, or opium and ipecac. It is an important remedy in diarrhœa and in the second stage of dysentery. I have tried it in one case of epidemic cholera with very unexpected success, after the failure of every thing else on which I had been accustomed to rely. The case was not one of the most rapid, but was of a kind which was generally found very unmanageable. It was attended by profuse, frequent, and exhausting rice-water discharges from the bowels, with occasional vomiting. A drachm of sugar of lead was given. It vomited and purged my patient actively, and speedy convalescence was the consequence. I have resorted to it occasionally, as an internal remedy, in gonorrhœa, with the best effects. It is believed it very rarely produces poisonous effects when used as a medicine; at least, it has never done so in my practice, though it has been commonly combined with opium, which is supposed to be an antidote to such effects.

